# Supplementary material for: Diagnostic Performance of Magnetic Resonance Imaging for Parathyroid Localization of Primary Hyperparathyroidism: A Systematic Review
Source: Diagnostics (Basel). 2023 Dec 22;14(1):25. doi: 10.3390/diagnostics14010025 (PMC10802165; doi:10.3390/diagnostics14010025)
Supplement: Supplementary file 1 [file diagnostics-14-00025-s001.zip › diagnostics-2752565-supplementary.pdf]

Supplementary Materials

Table S1: Full electronic search strategy performed in the PubMed-, and Embase- databases.

Table S2: QUADAS-2 assessment tool.

Table S3: Quality assessment using QUADAS-2 tool: Summary and separate outcome of risk of bias and concerns regarding applicability for included studies.

Table S1: Full electronic search strategy performed in the PubMed-, and Embase-databases.

#	Search Term	Hits
1	exp hyperparathyroidism/ or exp parathyroid tumor/ or 'hyperparathyroid*'.ti,ab,kf.	81563
2	exp parathyroid gland/ or 'parathyroid'.ti,ab,kf.	122170
3	exp adenoma/ or 'adenoma'.ti,ab,kf.	303758
4	2 AND 3	18152
5	1 OR 4	84465
6	exp nuclear magnetic resonance imaging/ or ('magnetic resonance imaging' or MRI).ti,ab,kf.	1784694
7	exp "Sensitivity and Specificity"/	1132287
8	Sensitivity.ti,ab,kf.	2266283
9	Specificity.ti,ab,kf.	1288433
10	accuracy.ti,ab,kf.	1252762
11	7 OR 8 OR 9 OR 10	4364373
12	5 AND 6 AND 11	471

Table S2: QUADAS-2 assessment tool.

QUADAS-2 is structured so that 4 key domains are each rated in terms of the risk of bias andthe concern regarding applicability to the research question (as defined above). Each key domain has a set of signalling questions to help reach the judgments regarding bias and applicability.

DOMAIN 1: PATIENT SELECTION

A. Risk of Bias

Describe methods of patient selection:

- ❖ Was a consecutive or random sample of patients enrolled? Yes/No/Unclear
- ❖ Was a case-control design avoided? Yes/No/Unclear
- ❖ Did the study avoid inappropriate exclusions? Yes/No/Unclear

Could the selection of patients have introduced bias? RISK: LOW/HIGH/UNCLEAR

B. Concerns regarding applicability

Describe included patients (prior testing, presentation, intended use of index test and setting)**:**

Is there concern that the included patients do not match the review question? CONCERN: LOW/HIGH/UNCLEAR

DOMAIN 2: INDEX TEST(S)

If more than one index test was used, please complete for each test.

A. Risk of Bias

Describe the index test and how it was conducted and interpreted:

- ❖ Were the index test results interpreted without knowledge of the results of the reference standard? Yes/No/Unclear
- ❖ If a threshold was used, was it pre-specified? Yes/No/Unclear

Could the conduct or interpretation of the index test have introduced bias? RISK: LOW /HIGH/UNCLEAR

B. Concerns regarding applicability

Is there concern that the index test, its conduct, or interpretation differ from the review question? CONCERN: LOW /HIGH/UNCLEAR

DOMAIN 3: REFERENCE STANDARD

A. Risk of Bias

Describe the reference standard and how it was conducted and interpreted:

- ❖ Is the reference standard likely to correctly classify the target condition? Yes/No/Unclear
- ❖ Were the reference standard results interpreted without knowledge of the results of the index test? Yes/No/Unclear

Could the reference standard, its conduct, or its interpretation have introduced bias? **RISK: LOW /HIGH/UNCLEAR**

B. Concerns regarding applicability

Is there concern that the target condition as defined by the reference standard does not match the review question? **CONCERN: LOW /HIGH/UNCLEAR**

DOMAIN 4: FLOW AND TIMING

A. Risk of Bias

Describe any patients who did not receive the index test(s) and/or reference standard or who were excluded from the 2x2 table (refer to flow diagram):

Describe the time interval and any interventions between index test(s) and reference standard:

- ❖ Was there an appropriate interval between index test(s) and reference standard? Yes/No/Unclear
- ❖ Did all patients receive a reference standard? Yes/No/Unclear
- ❖ Did patients receive the same reference standard? Yes/No/Unclear
- ❖ Were all patients included in the analysis? Yes/No/Unclear

Could the patient flow have introduced bias? **RISK: LOW /HIGH/UNCLEAR**

Table S3: Quality assessment using QUADAS-2 tool: Summary and separate outcome of risk of bias and concerns regarding applicability for included studies.

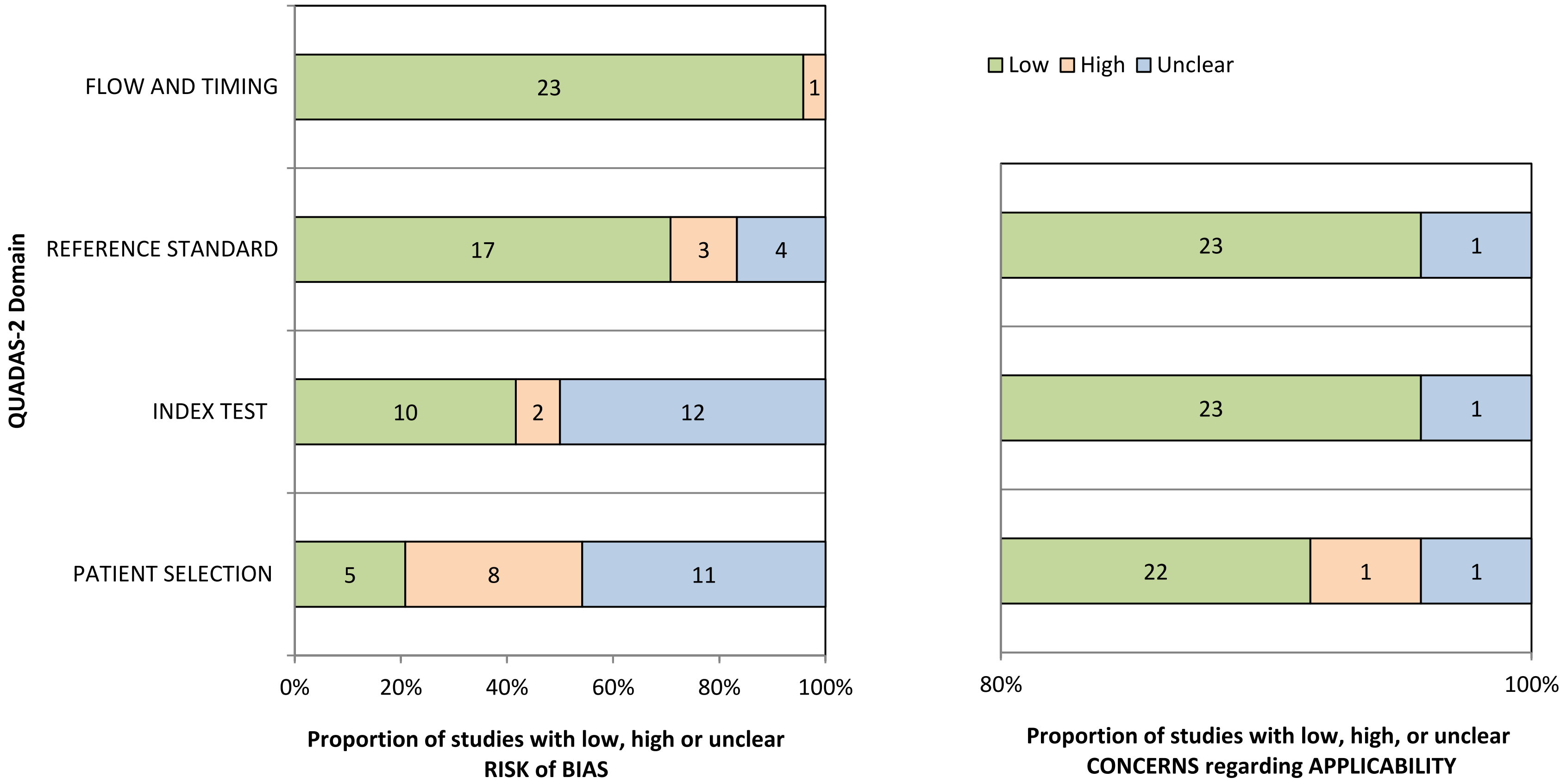

Study	Risk of Bias				Applicability Concerns		
	PATIENT SELECTION	INDEX TEST	REFERENCE STANDARD	FLOW AND TIMING	PATIENT SELECTION	INDEX TEST	REFERENCE STANDARD
Acar et al.	Unclear	Unclear	Low	Low	Low	Low	Low
Agha et al.	High	Low	Low	Low	Low	Low	Low
Akbaba et al.	Unclear	Unclear	Unclear	Low	Low	Low	Low
Araz et al.	Low	Unclear	High	High	Low	Low	Low
Argiro et al.	Low	Low	Low	Low	Low	Low	Low
Aschenbach et al.	High	Low	Low	Low	Low	Low	Low
Becker et al.	Unclear	Low	Low	Low	Low	Low	Low
Bijnens et al.	Low	Unclear	Low	Low	Low	Unclear	Low
Cakal et al.	Unclear	High	Unclear	Low	Low	Low	Low
Gotway et	Unclear	Unclear	Unclear	Low	High	Low	Unclear
Graves et al.	Low	Low	Low	Low	Low	Low	Low
Grayev et al.	Unclear	High	High	Low	Low	Low	Low
Hofer et al.	High	Unclear	Low	Low	Low	Low	Low
Khafif et al.	Unclear	Low	Low	Low	Low	Low	Low
Kluijfhout et al. (2017)	Low	Low	Low	Low	Low	Low	Low
Kluijfhout et al.(2016)	High	Unclear	Low	Low	Low	Low	Low
Memeh	High	Unclear	Low	Low	Low	Low	Low
Merchavy	Unclear	Low	Low	Low	Low	Low	Low
Michel	High	Unclear	Low	Low	Low	Low	Low
Murugan	Unclear	Unclear	Low	Low	Low	Low	Low
Ozturk et al.	Unclear	Low	Low	Low	Low	Low	Low
Ruf et al.	Unclear	Low	Low	Low	Unclear	Low	Low
Saeed let al.	High	Unclear	High	Low	Low	Low	Low
Sekiyama et al.	High	Unclear	Unclear	Low	Low	Low	Low
